# Coverage of intermittent preventive treatment of malaria in pregnancy (IPTp) influences delivery outcomes among women with obstetric referrals at the district level in Ghana

**DOI:** 10.1186/s12936-020-03288-4

**Published:** 2020-06-24

**Authors:** Mary Amoakoh-Coleman, Daniel K. Arhinful, Kerstin Klipstein-Grobusch, Evelyn K. Ansah, Kwadwo A. Koram

**Affiliations:** 1 Department of Epidemiology, Noguchi Memorial Institute for Medical Research, University of Ghana, Legon, Ghana; 2 Julius Global Health, Julius Center for Health Sciences and Primary Care, University Medical Center Utrecht, Utrecht University, Utrecht, Netherlands; 3 Division of Epidemiology and Biostatistics, School of Public Health, Faculty of Health Sciences, University of the Witwatersrand, Johannesburg, South Africa; 4 Center for Malaria Research, Institute of Health Research, University of Health & Allied Sciences, Ho, Ghana

**Keywords:** Obstetric referrals, Intermittent presumptive treatment of malaria in pregnancy, Coverage, Delivery outcomes, Maternal, Neonatal

## Abstract

**Background:**

The aim of the study was to determine the coverage of intermittent preventive treatment of malaria in pregnancy (IPTp) and its relationship with delivery outcomes among obstetric referral cases at the district level of healthcare.

**Methods:**

An implementation research within three districts of the Greater Accra region was conducted from May 2017 to February 2018, to assess the role of an enhanced inter-facility communication system on processes and outcomes of obstetric referrals. A cross-sectional analysis of the data on IPTp coverage as well as delivery outcomes for the period of study was conducted, for all the referrals ending up in deliveries. Primary outcomes were maternal and neonatal complications at delivery. IPTp coverage was determined as percentages and classified as adequate or inadequate. Associated factors were determined using Chi square. Odds ratios (OR, 95% CI) were estimated for predictors of adequate IPTp dose coverage for associations with delivery outcomes, with statistical significance set at p = 0.05.

**Results:**

From a total of 460 obstetric referrals from 16 lower level facilities who delivered at the three district hospitals, only 223 (48.5%) received adequate (at least 3) doses of IPTp. The district, type of facility where ANC is attended, insurance status, marital status and number of antenatal clinic visits significantly affected IPTp doses received. Adjusted ORs show that adequate IPTp coverage was significantly associated with new-born complication [0.80 (0.65–0.98); p = 0.03], low birth weight [0.51 (0.38–0.68); p < 0.01], preterm delivery [0.71 (0.55–0.90); p = 0.01] and malaria as indication for referral [0.70 (0.56–0.87); p < 0.01]. Positive association with maternal complication at delivery was seen but was not significant.

**Conclusion:**

IPTp coverage remains low in the study setting and is affected by type of health facility that ANC is received at, access to health insurance and number of times a woman attends ANC during pregnancy. This study also confirmed earlier findings that, as an intervention IPTp prevents bad outcomes of pregnancy, even among women with obstetric referrals. It is important to facilitate IPTp service delivery to pregnant women across the country, improve coverage of required doses and maximize the benefits to both mothers and newborns.

## Background

Without appropriate control interventions, 45% of pregnancies in malaria-endemic areas in sub-Saharan Africa are at risk of *Plasmodium falciparum* malaria [1]. Malaria in pregnancy (MIP) accounts for significant morbidity and mortality for pregnant women and their new-borns, especially in sub-Saharan Africa (SSA) [2]. It is globally a recognizable cause of stillbirths and neonatal deaths [3], with higher burdens in malaria endemic regions. A lot of interventions have been tested and prescribed to reduce the burden of MIP, and these have been deployed with varying effects on pregnancy outcomes depending on many factors including level of transmission and maternal factors like parity [1, 4]. These interventions include use of insecticide-treated bed nets and other materials, use of intermittent preventive treatment in pregnancy (IPTp) and effective case management (including testing and prompt treatment with highly effective drugs) [4, 5].

The use of IPTp is particularly useful in moderate to high transmission areas where many infected women are asymptomatic with resultant maternal anaemia and placental parasitaemia that leads to low birth weight, prematurity and intrauterine growth restriction [5–8]. Some reports show that IPTp coverages in SSA are not meeting national targets, with various reasons assigned for the trend [9, 10]. The reasons identified can be categorized as individual factors such as education and timing of antenatal clinic (ANC) visits, household level issues such as economic power to purchase medicines, including sulfadoxine-pyrimethamine (SP), and health system issues like stock-outs of SP, ANC user fees, poor counselling [11, 12]. The unavailability of SP at the health facility when women attend ANC leads to many missed opportunities.

**Fig. 1:**
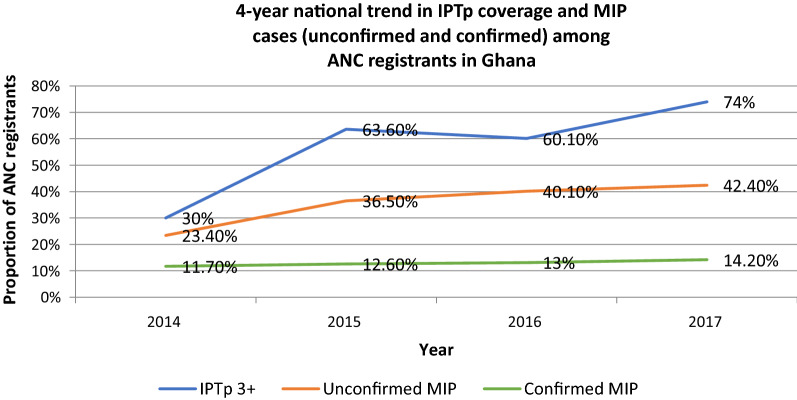
A 4-year national trend in IPTp coverage and Malaria in pregnancy (MIP) cases (unconfirmed and confirmed) among antenatal clinic (ANC) registrants in Ghana *Data source: NMCP, GHS (2014*–*2017)*

Malaria is endemic in Ghana with almost 50% of pregnant women at term (36–40 weeks gestation) and 12–36% of women at 32 weeks gestation and above, having asymptomatic malaria parasitaemia [13]. According to reports from the National Malaria Control Programme (NMCP), in 2012, 18.8% of all pregnancy related admissions were due to MIP while 3.4% of maternal deaths were due to MIP [14]. In Ghana, the use of IPTp has been shown to be an effective method for controlling MIP. In a study conducted in southern Ghana in 2006, after the implementation of IPTp from 2000, maternal anaemia had reduced by 33% and placental parasitaemia reduced by 57% in 2006 as compared to 2000 [15]. The NMCP promotes IPTp of malaria using the World Health Organization (WHO) recommended SP, for all pregnant women. This is taken monthly starting from quickening or 16 weeks of pregnancy through to delivery. From the onset of implementation of IPTp in Ghana, the required number of SP doses was three to be received between 4 to 6 months of gestation as per the WHO recommendation [16]. In 2016, a circular by the NMCP sought to amend the 2014 IPTp policy, requiring pregnant women attending antenatal care to receive up to 5 doses or more of SP until the time of delivery, provided that the doses are given at least one month apart. Despite high antenatal coverage, in 2012, only 44.4% of pregnant women seen at a district hospital received at least 3 doses of IPTp [8]. The NMCP’s national records show that the proportion of ANC registrants who received at least 3 doses of IPTp increased from 30% in 2014 to 74% in 2017 [17]. However, proportion of both unconfirmed and confirmed cases of MIP among ANC registrants also increased over the same period (Fig. 1). This trend could be due to increased testing for malaria in over the period.

Availability of SP is one key factor affecting IPTp coverage in Ghana. Stock-out of SP has been common in Ghana, with patients in such cases either not receiving any doses at the health facility or at best given a prescription to purchase the medicine at a pharmacy.

Obstetric complications are prevalent in Ghana with many resultant referrals from lower levels of care to higher levels. Co-morbidities such as malaria in pregnancy and its complications such as anaemia potentially could affect outcomes of some obstetric complications. Since there is a public health intervention such as administration of SP during pregnancy to address MIP, it is important that women receive adequate doses to protect them. The aim of the study was to determine the coverage of intermittent preventive treatment of malaria in pregnancy (IPTp) and its relationship with delivery outcomes among a cohort of obstetric referrals at the district level of healthcare.

## Methods

### Design and setting

At delivery, a cross-sectional analysis of data for obstetric referrals which ended in deliveries in three districts/municipalities in the Greater Accra region of Ghana from May 2017 to January 2018 was conducted. This study was part of an implementation research to evaluate the role of an enhanced inter-facility communication system on the processes and outcomes of obstetric referrals in these districts in the region.

The Greater Accra region hosts Ghana’s capital city and has 20 administrative metropolises, municipalities, districts and sub-metropolises. It is mostly urban but has 4 rural districts. The study was conducted in Ga West which is semi-urban and Ada East and Ningo-Prampram which are largely rural. Two of the selected districts (Ada East and Ga West) have district hospitals while one (Ningo-Prampram) has a polyclinic as the highest-level public facility. It however has a private hospital where patients are referred to, which is included in this study. A small number of patients are also referred outside the district, (but were not included in the sample).

### Participants

Participants were pregnant women who had been referred from lower levels of care (health center, polyclinic, community clinic and community health and planning services [CHPS] compounds), within the district to the district hospital, and received care at the district hospital, over the period from May 2017 to January 2018, and delivered before discharge. These women, who were already part of a larger study, were included in this study because It was possible to analyse the pregnancy outcome as well as the IPTp doses and other related factors at one point in time without the need to follow them up.

### Data collection and variables

A facility audit was conducted to, among other things, assess the availability of sulfadoxine-pyrimethamine (SP) at pharmacy or dispensary as well as stock-out of SP at the pharmacy or dispensary within 6 months prior to and at the time of the study. The availability of a laboratory that offers malaria testing services was determined. Participants answered a questionnaire while on admission, and their records as well as hospital registers were also reviewed during the period of stay in hospital for additional data using a checklist. Data was collected on participants’ sociodemographic characteristics, previous pregnancy history, current pregnancy details, indication for referral and delivery factors. The primary outcome variables were any maternal complication at delivery and any new-born complications at delivery. Secondary outcomes were gestational age, birth weight and anaemia at delivery, gender of baby and whether malaria was the indication for the referral. Focus was not on complications during the whole pregnancy period because participants had been referred and most of them had one complication or the other before delivery.

Independent variables were dose of IPTp received during pregnancy and others shown in Table 1. For analysis of relationship between IPTp coverage and delivery outcomes, IPTp doses received was categorized as adequate or inadequate based on the WHO recommendation of at least 3 doses during pregnancy [16],

**Table 1:** Definition of variables for the study

Facility factors	Individual factors	Pregnancy and referral factors	Delivery factors
District	Age	Parity	Sex of baby: *male, female*
Type of facility where ANC was received: *Health centre, polyclinic, community clinic, CHPS*	Health Insurance coverage: *Yes or No*	Gestational age (Trimester) at first ANC visit: *1st, 2nd, rd*	Gestational age at delivery: *preterm (*<* 37* *weeks)* *Term (37*–*40* *weeks)* *Post term (*>* 40* *weeks)*
Ownership of facility: *Government or Private*	Age	Number of ANC visits during pregnancy: *0, 1*–*3, 4*+	Any new-born complication at delivery: *Yes or No*
Availability of sulfadoxine-pyrimethamine at pharmacy or dispensary: *Yes or No*	Highest educational attainment: *none, primary, secondary, tertiary*	History of previous pregnancy complication: *Yes or No*	Any maternal complication at developed at delivery: *Yes or No*
Stock-out of sulfadoxine-pyrimethamine at pharmacy or dispensary within 6 months prior to study: *Yes or No*	Marital status: *single, married, living together*	Indication for referral is Malaria: *Yes or No*	Haemoglobin level at delivery: *Normal (≥ 12* *g/dl), mild anaemia (10.0*–*11.9* *g/dl),* *moderate anaemia (9.9*–*7.0* *g/dl)* *severe anaemia (*<* 7.0* *g/dl)*
	Employment status: *Yes or No*	Indication for referral is a pregnancy complication: *Yes or No*	Birth weight of baby: *Low birth weight (*< *2.5* *kg)* *Normal weight (2.5*–*4.0* *kg)* *Big baby (*>* 4.0* *kg)*
		Dose of IPTp received during pregnancy: *0, 1*–*3, 4*–*5, *> *5;* *Adequate (NMCP/WHO): (5 *+*)/(3 *+*),* *Inadequate (NMCP/WHO): (*< *5)/(*<* 3)*	

*ANC* antenatal care, *IPTp* intermittent presumptive treatment of malaria in pregnancy

### Statistical analysis

Total missing data were more than 5% and this level of missing data is rarely random. Thus, multiple imputations of missing data were conducted and analysis was based on the imputed data set. Descriptive analysis using frequencies and proportions was done for all the independent and outcome variables. Bivariate analysis using χ2 test was used to investigate the relationship between the IPTp dose coverage and other independent variables, with detection of significance set at p < 0.05. Using a backward stepwise approach, a model was built with all the significant factors in the χ2 analysis to determine the predictors of IPTp dose coverage. For analysis of association between IPTp coverage and outcomes, IPTp doses received was categorized as adequate or inadequate based on the WHO recommendation of at least 3 doses during pregnancy [16]. Associations between IPTp dose coverage and the outcomes were estimated using odds ratios (OR), with their 95% confidence intervals (CI). Significance tests were based on Wald Chi square tests and p-values < 0.05 were considered significant, with all potential confounders adjusted for. Data analysis was carried out using IBM SPSS Statistics for Windows, Version 20.0. Armonk, NY: IBM Corp.

### Strength and limitations

The strength of this paper is in the fact that it focused on women with obstetric referrals and not all pregnant women in general as most studies have looked at. Most of these women have some pregnancy complication and yet the data shows that adequate IPTp coverage is associated with improved delivery outcomes among them. Patient records for data on the IPTp coverage and some of the secondary outcome variables were used, leading to some missing data. This is a limitation of the study which was addressed by doing multiple imputation of data for the analysis. Also, it must be noted that this work was done among referred pregnant women and so results may not reflect what the situation is for the entire pregnant women population.

## Results

A total of seven hundred and fifty-three (753) obstetric referrals were attended to in the three district hospitals over the nine-month period of the study, from sixteen (16) lower level facilities within the districts. Out of these, five hundred and thirty-nine (539) delivered at the hospital before discharge, and of these, four hundred and sixty (460) had data on IPTp coverage and were included in the analysis for this paper. The lower level facilities included one polyclinic, eight health centres, eight CHPS compounds and two community clinics. Apart from one hospital and one clinic which were privately owned, the other facilities were all government owned. All the 16 facilities from where women received ANC had stocks of SP at the beginning of the study and had experienced no stock outs of same within the past 6 months prior to the start of the study. Nine out of the sixteen facilities had laboratories and each of them offered malaria testing services. Most of the participants received ANC at health centres (81.5%) and most of them came from one municipality (66.1%) mainly because it has and serves a bigger population.

### Characteristics of participants

The mean age (SD) of participants was 28.0 (6.5) years, with most of them (73.3%) within the 20–35 years category. Majority of the women had 0–3 parity (71.6%), primary/basic level education (63.7%), were employed (75.7%) and either married or living together with their partners (84.8%). Almost all (98.7%) of the women, had valid national health insurance coverage.

First ANC visit for most women was in either first or second trimester (49.3% and 42.7% respectively). The mean gestational age (SD) at first ANC visit was 3.7 (1.8) months and the mean gestational age at referral (SD) at 35.5 (7.9) weeks, which is in the third trimester. Most of the women had at least 4 ANC visits. Although only 13.5% had a positive history of previous pregnancy complication, 61.7% of them had had a complication in the current pregnancy. The reason for the current referral was due obstetric complication in 82.6% of women, with malaria in pregnancy being the indication for 1.3% of them. Table 2 shows the proportion of all the variables studied among participants.

**Table 2:** Baseline characteristics of women in the study

Variable	Category	Frequency N = 460	%	
District	A	94	20.5	
	B	62	13.5	
	C	304	66.1	
Ownership	Government	398	86.5	
	Private	62	13.5	
Covered by Health Insurance	Yes	454	98.7	
Age category	< 20	58	12.6	
	20–35	339	73.7	
	> 35	63	13.7	
Parity category	0	147	32.0	
	1–2	182	39.6	
	3–4	103	22.4	
	> 4	28	6.1	
Highest education attained	None	71	15.4	
	Primary/Basic	293	63.7	
	Secondary/Vocational	83	18.0	
	Tertiary	10	2.2	
Marital status	Single	70	15.2	
	Married	251	54.6	
	Living together	139	30.2	
Employed	Yes	348	75.7	
	No	112	24.3	
Trimester at first ANC visit	1st	227	49.3	
	2nd	197	42.7	
	3^rd^	33	7.2	
No. of ANC visits so far	0	18	3.9	
	1–3	96	20.9	
	≥ 4	344	74.8	
Trimester at referral	1st	22	4.8	
	2nd	22	4.8	
	3rd	410	89.1	
Previous pregnancy complication	Yes	62	13.5	
	No	398	86.5	
Complication in current pregnancy	Yes	284	61.7	
Previous referral in current pregnancy	Yes	12	2.6	
Type of referring facility/where ANC and IPTp were received	Health center	375	81.5	
	Polyclinic	38	8.3	
	Clinic	38	6.1	
	CHPS	19	4.1	
Is referral emergency	Yes	243	52.8	
Reason for referral	Complication	380	82.6	
	Lack of required staff	79	17.2	
	Lack of space/bed	3	0.7	
	Lack of logistic/supplies/infrastructure	36	7.8	
	Lack of required service	28	6.1	
Clinical Indication (complication) for this referral	Anemia	50	10.87	
	Vaginal bleeding/APH	21	4.6	
	Fetal distress	10	2.2	
	IUFD/No fetal heart	13	2.8	
	Malaria in pregnancy	6	1.3	
	Malpresentation	19	4.1	
	PIH/Preeclampsia/Eclampsia	82	17.8	
	Post date	60	13.0	
	Postpartum hemorrhage	6	1.3	
	Previous caesarean section	40	8.7	
	Prolonged labor	29	6.3	
	Other	79	17.2	
IPTp Doses received by delivery	0	90	19.6	
	1–3	279	60.7	
	4–5	83	18.0	
	> 5	6	1.3	
	< 5 (Inadequate, NMCP)	420	91.3	
	≥ 5 (Adequate, NMCP)	40	8.7	
	< 3 (Inadequate, WHO)	237	51.5	
	≥ 3 (Adequate, WHO)	223	48.5	
Gestational maturity at delivery	Pre term	63	13.7	
	Term	288	62.6	
	Post maturity	109	23.7	
Hemoglobin at delivery	Normal	78	17.0	
	Mild anemia	194	42.2	
	Moderate anemia	188	40.8	
Maternal complication developed at delivery	Yes	182	39.6	
	Anemia	12	2.6	
	Abruptio placenta	7	1.5	
	PIH/eclampsia	33	7.2	
	Prolonged/obstructed labor	48	10.4	
	Malpresentation	32	7.0	
	Postpartum hemorrhage	8	1.7	
New-born complication	Yes	87	18.9	
	Low Birth Weight	38	8.3	
	Fetal distress	25	5.4	
	Asphyxia	18	3.9	
	Stillbirth	6	1.3	
	Intrauterine death	2	0.4	
	Sepsis	12	2.8	
	Jaundice	1	0.2	
Birth weight category	Low birth weight	39	8.5	
	Normal	397	86.3	
	Big baby	10	2.2	
Sex of baby	Male	253	54.9	
	Female	207	45.1	

Variable	Mean (95% CI)	Standard deviation	Median	Inter-quartile range
Age of woman (years)	28.0 (27.4 –28.6)	6.5	28.0	10.0
Gestational age at first ANC (months)	3.7 (3.5–3.9)	1.8	3.0	3.0
Gestational age at referral (weeks)	35.5 (34.7–36.2)	7.9	38.0	5.0
Gestational age at delivery (weeks)	38.4 (38.0–38.8)	3.8	39.0	2.0
Hemoglobin at delivery (g/dl)	10.0 (9.9–10.2)	1.6	10.1	2.5
Birth weight (kg)	3.1 (3.05–3.14)	0.5	3.1	0.6
Doses of IPTp received	2.2 (2.1–2.4)	1.5	2.0	2.0

**Fig. 2:**
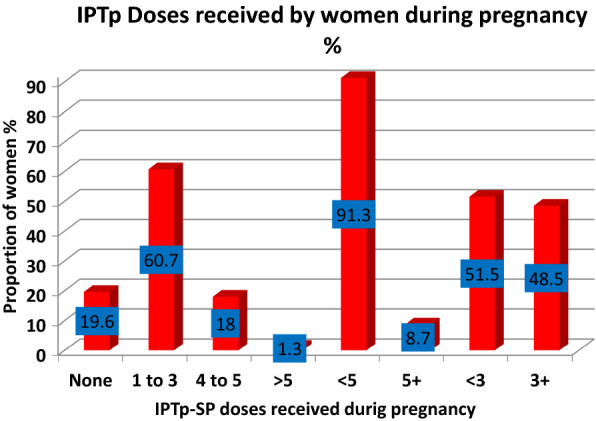
A bar graph showing proportion of referred women receiving different doses of IPTp–SP during pregnancy in the three districts

Most women (60.7%) received up to 3 doses (1–3) of IPTp during pregnancy, but when IPTp doses received was categorized as adequate or inadequate based on the NMCP recommendation of at least 5 doses from 2016, only 40 (8.7%) of the women received adequate dose of at least 5 doses during pregnancy. When IPTp doses received was categorized as adequate or inadequate based on the WHO recommendation of at least 3 doses during pregnancy (16), only 223 (48.5%) of the women received adequate doses of IPTp. Figure 2 shows the distribution of women across different doses of IPTp coverage.

### Predictors of IPTp dose coverage

Bivariate analysis showed significant differences between IPTp dose coverage for most of the independent variables (Table 3). However, when adjusted for other factors in a multivariable analysis, only the variables district, type of facility where ANC was received, health insurance coverage, marital status and number of ANC visits were significantly associated with IPTp dose coverage (Table 4).

**Table 3:** Factors associated with different IPTp dose coverages among participants

USING WHO CRITERIA		IPTp coverage		
Variable	Category	Inadequate (< 3)	Adequate (+3)	*p* value
District	A	42.6	57.4	< *0.01*
	B	46.8	53.2	
	C	55.3	44.7	
Facility type	Health centre	51.5	44.7	< *0.01*
	Polyclinic	42.9	57.1	
	Community clinic	50.0	50.0	
	CHPS	68.4	31.6	
Ownership	Government	52.3	47.7	*0.05*
	Private	46.8	53.2	
Insurance	Yes	51.8	48.2	*0.03*
	No	33.3	66.7	
Age	< 20	51.7	48.3	*0.58*
	20–35	51.0	49.0	
	> 35	54.0	46.0	
Parity	0	52.4	47.6	< *0.01*
	1–2	55.5	44.5	
	3–4	46.6	53.4	
	> 4	39.3	60.7	
Education	None	49.3	50.7	< *0.01*
	Primary/Basic	52.6	47.4	
	Secondary	45.8	54.2	
	Tertiary	80.0	20.0	
Marital status	Single	44.3	55.7	< *0.01*
	Married	49.4	50.6	
	Living together	59.0	41.0	
Employed	Yes	52.3	47.7	*0.15*
	No	49.1	50.9	
Trimester at first ANC visit	1st	53.7	46.3	*0.11*
	2nd	50.0	50.0	
	3rd	48.5	51.5	
Number of ANC visits	0	50.0	50.0	*0.67*
	1–3	53.1	46.9	
	4 +	51.2	48.8	
Previous pregnancy complication	Yes	53.4	46.6	*0.44*
	No	51.2	48.8	

Predictors of adequate IPTp doses received during pregnancy				
Predictors	Category	Adjusted OR (95% CI)	*p*-value	
District	A	1.41 (1.14–1.74)	< *0.01*	
	B	1.18 (0.84–1.65)	*0.34*	
	C	¥		
Type of Health Facility	Health center	1.90 (1.22–2.97)	*0.01*	
	Polyclinic	1.74 (1.03–2.93)	*0.04*	
	Clinic	2.77 (1.60–4.79)	< *0.01*	
	CHPS	¥		
Highest Education attained	None	¥		
	Primary/basic	1.00 (0.80–1.26)	*0.99*	
	Secondary	1.44 (1.09–1.92)	*0.01*	
	Tertiary	0.41 (0.21–0.81)	*0.01*	
Insurance coverage	Yes	0.24 (0.09–0.66)	*0.01*	
	No	¥		
Marital status	Single	¥		
	Married	0.86 (0.66–1.13)	*0.28*	
	Living together	0.66 (0.50–0.86)	< *0.01*	
Number of ANC visits	0	0.57 (0.35–0.93)	*0.02*	
	1–3	0.86 (0.71–1.06)	*0.15*	
	4+	¥		

*ANC* antenatal clinic, *CHPS* Community based Health Planning and Services, *WHO* World Health Organization, *¥* Reference category

**Table 4:** Association of pregnancy outcomes with adequate dose of IPTp received and predictors of adequate IPTp doses received during pregnancy

Outcome	Category	Unadjusted OR (95% CI)	*p*-value	Adjusted OR^a^ (95% CI)	*p*-value
New-born complication	Yes	0.74 (0.61–0.90)	**< *0.01***	0.80 (0.65–0.98)	***0.03***
Maternal complication developed at delivery	Yes	0.71 (0.61–0.83)	**< *0.01***	0.88 (0.72–1.07)	***0.20***
Anaemia at delivery	Normal	¥ (reference)		¥	
	Mild	1.11 (0.89–1.34)	*0.36*	1.14 (0.90–1.44)	*0.29*
	Moderate	0.91 (0.77–1.14)	*0.41*	0.90 (0.71–1.14)	*0.38*
Hemoglobin level		0.93 (0.83–0.99)	***0.03***		
Birth weight	LBW	0.55 (0.41–0.72)	**<*0.01***	0.51 (0.38–0.68)	**<*0.01***
	Normal	¥		¥	
	Big baby	0.98 (0.58–1.63)	*0.92*	0.38 (0.20–0.72)	**<*0.01***
Term at gestation	Preterm	0.72 (0.57–0.91)	***0.01***	0.71 (0.55–0.90)	***0.01***
	Term	¥		¥	
	Post term	1.06 (0.88–1.27)	*0.56*	0.95 (0.77–1.61)	*0.59*
Sex of baby	Female	0.89 (0.77–1.04)	*0.14*	1.13 (0.96–1.33)	*0.14*
	Male	¥		¥	
Referral is due to malaria	Yes	0.71 (0.34–1.49)	*0.36*	0.70 (0.56–0.87)	**<*0.01***
	No	¥		¥	

*ANC* antenatal clinic, *CHPS* Community-based Health Planning and Services, *LBW* low birth weight

^a^Adjusted for district, facility type where ANC was received, insurance coverage, age, parity, educational level, marital status, employment status, trimester for first ANC visit, number of ANC visits in pregnancy, pregnancy complication is reason for referral and history of previous pregnancy complication

### IPTp dose coverage and delivery outcomes

One hundred and eighty-two (39.6%) of the participants had at least one maternal complication developed at delivery, and eighty-seven (18.9%) of their new-borns had at least one complication. Maternal complications at delivery included pre-eclampsia and eclampsia (7.2%), obstructed/prolonged labour (10.4%) and post-partum haemorrhage (1.7%). New-born complications included low birth weight (8.3%), asphyxia (3.9%), still birth (1.3%) and sepsis (2.8%), (Table 2). With respect to associations with delivery outcomes, after adjusting for potential confounders such as district, facility type where ANC was received, insurance coverage, age, parity, educational level, marital status, employment status, trimester for first ANC visit, number of ANC visits in pregnancy, pregnancy complication is reason for referral and history of previous pregnancy complication, adequate IPTp coverage showed significant associations with prevalence of new-born complication [0.80 (0.65–0.98); p = 0.03], low birth weight [0.51 (0.38–0.68); p < 0.01], big baby [0.38 (0.20–0.72); p < 0.01], preterm delivery [0.71 (0.55–0.90); p = 0.01] and malaria as indication for referral [0.70 (0.56–0.87); p < 0.01]. Adequate IPTp dose coverage was positively associated with maternal complication at delivery, but this association was not significant after adjusting for confounders [0.88 (0.72–1.07); p = 0.20] (Table 4).

## Discussion

### Main findings

This study explored the relationship between IPTp dosage coverage and its relationship with delivery outcomes among obstetric referrals at the district healthcare. Only 48.5% of participants received adequate (at least 3) doses of IPTp during pregnancy, although 74.8% of them attended ANC at least four times. Proportion of women receiving at least 5 doses was 8.7%. Doses of IPTp received was determined by the district the IPTp was received in, type of facility, marital status, health insurance coverage, and the number of ANC visits during pregnancy. For the study population, a significant association was found between prevalence of new-born complications (including low birth weight, preterm delivery) and the doses of IPTp received. Adequate IPTp dose coverage was not significantly associated with maternal complication at delivery. These findings are consistent with various studies in similar context [18–23].

### Implications for obstetric care and policy

From the results, less than 50.0% of the participants received a minimum of 3 doses of IPTp as per WHO recommendation, while less than 10.0% received minimum of 5 doses as per the Ghana NMCP recommendation. Available evidence suggests low and inter and intra country variations in levels of IPTp utilization in Africa, especially in high malaria transmission areas and despite the availability of an IPTp policy and high ANC attendance [19, 24, 25].

Kenya was one of the countries to have started IPTp implementation early on, and had a national coverage of 80.4% as at 2013, while its counterpart country of Malawi had 35.5% coverage [24]. Comparative analysis of 2014 to 2016 malaria indicator survey (MIS) results from some sub-Saharan African countries showed Ghana as having the highest coverage for 3plus doses of IPTp (60.0%) and Mali with the lowest coverage (11.5%) [25]. In Ghana, institutional data shows that IPTp coverage increased from 30.0% in 2014 to 74.0% in 2017, with the coverages in Greater Accra region where this work was done improving from 32.4% to 79.0% over the same period [17]. Thus, the coverage estimated in this study for participants who had been seen within the three districts is below both the national and regional coverages. While the WHO encourages countries to adapt its IPTp policy to suit individual country contexts, it is important for Ghana to deliberate on whether it still wants to pursue a minimum of five doses of IPTp as a country, and if so, put in the necessary framework to ensure that it is attainable, since 8.7% is woefully inadequate. The national and regional coverages for 5 doses in 2017 are similar, being 8.9% and 9.6% respectively [17]. With many countries in the subregion still recommending a minimum of 2 or 3 doses [25–28] and the MIS also assessing minimum of 3 doses, perhaps Ghana should probably stick to the minimum of 3 doses stipulated in the 2014 policy and work on improving coverage for that.

Various factors have been cited as influencing IPTp coverages across different settings, and these include health system factors [24]. Consistent with other studies, this study found that district and type of facility where ANC was received were significantly associated with IPTp dose coverage [20, 24]. The district, depending on whether it is rural or urban depicts the type of health facility and consequently the capacity of the health system at that level. Two of the study sites were rural while one was semi-urban. One key factor related to health systems is the supply of medicines, in this case SP for IPTp. In this study, none of the facilities had stockouts of the medicine both at the time of the study or 6 months prior to that. Perhaps the use of recommended 5 doses per woman to project procurement of SP as contained in the NMCP circular is contributing to the availability of the medicine. One possible reason for variation in coverage between districts and facility type could be the fact that in Ghana, administration of IPTp is encouraged to be under direct observation by the health worker. Ability of health workers to do this consistently will likely influence coverage and documentation of IPTp administered. Unfortunately, we did not explore this as this study was carried out at delivery. Providers need to be encouraged and monitored to deliver the medicine to women consistently to increase uptake. Quality improvement processes such as supportive supervision has been prescribed to improve IPTp coverage [24, 29].

Beyond availability of SP, another health system factor that has been found to influence IPTp coverage is leadership and governance [30]. Leadership makes the provider or health system feel responsible for the delivery of the IPTp service, so that when set targets are not achieved, it is seen as provider or health system underachievement which needs to be addressed urgently. Leadership also ensures that health workers are well trained on the IPTp delivery policy, so that they appreciate the required targets and the scheduling of the doses for ANC attendants and work to achieve them.

Majority (98.7%) of study participants had functional health insurance coverage, but contrary to expectation, this study showed that, having insurance coverage has a significant negative association with IPTp coverage. Health insurance coverage, also a health system factor, increases access to health care. The financial barrier to accessing health services is removed and pregnant women can have the needed care, including IPTp services. Although this fact has been established in earlier studies [24, 31], this study could not confirm that. It will be useful if future studies can examine this finding to understand why and or validate it.

The number of times ANC is attended during pregnancy was positively associated with IPTp coverage, as has been found in other studies [19, 21, 25]. Dosing of IPTp for a pregnant woman needs planning around her ANC visits. Women should also be educated on the dangers of malaria in pregnancy and how the use of IPTp is protective as this has been found to increase IPTp uptake [27]. Evidence exists that when women are well informed about the IPTp service at the onset of ANC and when they are due for the doses, they will demand for it appropriately [23]. Having such high level of ANC attendance of over 98.0% in Ghana [31], with over 87.0% attending at least four times, health providers need to work with clients to optimize the benefits of frequent ANC attendance and avoid missed opportunities for IPTp services. Whereas being married was not significantly associated with IPTp coverage, living together with one’s partner showed a significant negative relationship with adequate IPTp coverage. There is the potential benefit of social support from the participants’ partners in showing care and following up to make sure pregnant women have ANC visits and adhere to care including medication. However, this benefit is not demonstrated in this study population.

This study has confirmed earlier findings that implementation of IPTp using SP remains effective in preventing the adverse consequences of malaria on maternal and fetal outcomes [19, 32]. This study did not demonstrate significant effect on maternal delivery outcomes among this group, and this could be because the women included in the study already had pregnancy complications for which they had been referred, some of which were related to delivery. However, the study demonstrates that adequate doses of IPTp reduces the odds that the reason for an obstetric referral was due to malaria in pregnancy as established by others [22]. Also, the results showed that odds of having low birth weight, preterm delivery and new-born complication in general was reduced among women who received adequate IPTp doses. Despite the fact that the effectiveness of IPTp in reducing neonatal morbidities among pregnancies has been established by other studies [33, 34], this study further showed that irrespective of the presence of other pregnancy complications for which women were referred, adequate doses of IPTp received by the time of delivery still remains protective for the new-born.

Preterm delivery with associated low birth weight accounts for 28.8% of neonatal mortality in Ghana [35]. The Ghana DHS 2014 estimated neonatal mortality as 29/1000 live births [30], and the target is to reduce this to 12/1000 live births per the sustainable development goal (SDG) target 3.2 [36, 37]. This requires putting in the required effort and resources into proven interventions such as use of IPTp and insecticide treated nets by pregnant women, together with skilled attendance at delivery. The group of women in the study can be said to be among the population at most need because they are already at risk of poor obstetric outcome because of the complications that resulted in their referral. If the IPTp intervention may help alleviate this risk, efforts should be made for them and all other pregnant women to be provided with adequate doses.

## Conclusions

Coverage of IPTp doses is influenced by type of health facility that ANC is received at, access to health insurance and number of times a woman attends ANC during pregnancy. Among women with obstetric referrals, adequate doses of IPTp significantly protect the mother from malaria in pregnancy as well and the baby from preterm delivery and its associated low birth weight. Continuous engagement within the health sector and with health partners, including the communities where pregnant women come from should be strengthened to facilitate IPTp service delivery to pregnant women, improve coverage of required doses and maximize the benefits to both mothers and new-borns.

## Data Availability

Data set for this paper is part of a bigger data set from the big study conducted and is currently stored on internal storage systems of NMIMR. We are able to provide data specific to this paper on request, once the purpose for the request fits into the ethics approval we received for the work. Request for the data set specific to this paper may be made to the NMIMR through the corresponding author (menba19@yahoo.com). Authors will still be working on the bigger data set to answer other questions and objectives of the bigger study so are unable to make it available to others as at now.

## Abbreviations

ANC: Antenatal clinic
CHPS: Community-based health and planning services
DHS: Demographic and Health Survey
GHS: Ghana Health Service
IPTp: Intermittent preventive treatment of malaria in pregnancy
LBW: Low birth weight
MIP: Malaria in pregnancy
NMCP: National Malaria Control Programme
SDG: Sustainable Development Goal
SSA: Sub-Saharan Africa
WHO: World Health Organization
